# Sleep Apnea and the Risk of Dementia: A Population-Based 5-Year Follow-Up Study in Taiwan

**DOI:** 10.1371/journal.pone.0078655

**Published:** 2013-10-24

**Authors:** Wei-Pin Chang, Mu-En Liu, Wei-Chiao Chang, Albert C. Yang, Yan-Chiou Ku, Jei-Tsung Pai, Hsiao-Ling Huang, Shih-Jen Tsai

**Affiliations:** 1 Department of Healthcare Management, Yuanpei University, HsinChu, Taiwan; 2 Department of Psychiatry, Kaohsiung Veterans General Hospital, Kaohsiung, Taiwan; 3 Department of Clinical Pharmacy, School of Pharmacy, Taipei Medical University, Taipei, Taiwan; 4 Department of Psychiatry, Taipei Veterans General Hospital, Taipei, Taiwan; 5 Division of Psychiatry, School of Medicine, National Yang-Ming University, Taipei, Taiwan; 6 Nursing Department, Kaohsiung Veterans General Hospital, Kaohsiung, Taiwan; Institute of Health Science, China

## Abstract

**Background:**

Sleep apnea (SA) has been associated with cognitive impairment. However, no data regarding the risk of dementia in patients with SA has been reported in the general population. This retrospective matched-control cohort study was designed to estimate and compare the risk of dementia in SA and non-SA patients among persons aged 40 and above over a 5-year period follow-up.

**Methods:**

We conducted a nationwide 5-year population-based study using data retrieved from the Longitudinal Health Insurance Database 2005 (LHID2005) in Taiwan. The study cohort comprised 1414 patients with SA aged 40 years who had at least 1 inpatient service claim or 1 ambulatory care claim. The comparison cohort comprised 7070 randomly selected patients who were matched with the study group according to sex, age, and index year. We performed Cox proportional-hazards regressions to compute the 5-year dementia-free survival rates after adjusting for potentially confounding factors.

**Results:**

The SA patients in this study had a 1.70-times greater risk of developing dementia within 5 years of diagnosis compared to non-SA age- and sex-matched patients, after adjusting for other risk factors (95% confidence interval (CI) = 1.26-2.31; *P* < .01). For the gender-dependent effect, only females with SA were more likely to develop dementia (adjust HR: 2.38, 95% CI =1.51–3.74; *P* < .001). For the age-dependent effect of different genders, males with SA aged 50-59 years had a 6.08 times greater risk for developing dementia (95% CI = 1.96-18.90), and females with SA aged ≥ 70 years had a 3.20 times greater risk of developing dementia (95% CI =1.71–6.00). For the time-dependent effect, dementia may be most likely to occur in the first 2.5 years of follow-up (adjusted HR:2.04, 95% CI =1.35-3.07).

**Conclusions:**

SA may be a gender-dependent, age-dependent, and time-dependent risk factor for dementia.

## Introduction

Sleep apnea (SA), which is one of the most common forms of sleep-disordered breathing, is characterized by abnormal pauses in breathing or instances of abnormally low breathing during sleep. In addition to dysfunctions of the endocrinal, cardiovascular, and metabolic systems, research has shown that SA can negatively affect functioning in multiple cognitive domains, such as attention and memory [1]. The cognitive deterioration of SA patients is age-dependent. Two studies have shown that middle-aged adults with severe SA are far more likely to have cognitive impairment than younger adults with equally severe SA [2,3]. A recent meta-analysis of 42 studies shows that patients with SA are significantly more impaired than healthy controls in verbal episodic memory (immediate recall, delayed recall, learning, and recognition) and visuospatial episodic memory (immediate and delayed recall), but not visual immediate recall or visuospatial learning [4]. 

Based on the cognitive impairment apparent in some SA patients and evidence suggesting higher rates of coexisting SA in some dementia patients [5–7], researchers have long suspected links between SA and dementia. In a recent prospective study of 298 women without dementia, Yaffe et al [8] found that older SA women had an increased risk of developing mild cognitive impairment or dementia after 5 years. They stressed the key role of SA in long-term memory impairment. Because this study involved mostly white, older women (mean age: 82.3 years), these findings may not be generalizable to men, younger populations, or more ethnically diverse populations. When only dementia was analyzed, the results of that study were consistent with the combined analysis, but show a reduced power to detect a significant difference between women with SA who develop dementia and women without SA who develop dementia [odds ratio 1.70; 95% confidence interval [CI] 0.88-3.27]. Thus far, data regarding the epidemiological association between SA and the development of dementia in a general population are still lacking. We hypothesized that SA patients may be at a greater risk of developing dementia. Because a low prevalence of dementia exists in the general population, we conducted a nationwide population study to investigate the relationship between SA and the subsequent development of dementia. This study also tested whether SA is a gender-dependent, age-dependent, and time-dependent risk factor for dementia.

## Materials and Methods

### Database

Taiwan began the National Health Insurance (NHI) program in 1995. The NHI program was mandatory, and covered nearly 99% of Taiwanese population of almost 23.5 million residents. The data set adopted in this study was obtained from the Longitudinal Health Insurance Database 2005 (LHID2005) from the National Health Insurance Research Database (NHIRD), which is managed by the Taiwanese National Health Research Institutes (NHRI). These claims data include registration and medical claims for 1 000 000 randomly sampled patients from all NHRI beneficiaries. The data set contained the entire medical claims data of 1 million beneficiaries from 1996 to 2010. According to the NHRI, there are no statistical differences in age and sex between the sampled group and all enrollees. To verify the accuracy of claim data, the Bureau of National Health Insurance (BNHI) performs quarterly expert reviews on a random sample of every 50 - 100 ambulatory and inpatient claims in each hospital and clinic. False reports of diagnoses result in a severe penalty from the BNHI.

Because the LHID2005 comprises secondary data (that cannot be used to identify patients) released to the public for research purposes, this study was exempt from full review by the Institutional Review Board. The LHID2005 data set is one of the largest nationwide population-based databases in the world, and many scientific studies have used its data.

### Study Population

The study cohort consists of SA patients aged 40 years or older who were newly diagnosed with SA [ICD-9-Codes 327.23, 780.51, 780.53, 780.57] between January 2003 and December 2005. For data accuracy, we included only cases in which patients obtained ≥2 SA diagnoses in outpatient visits or ≥1 inpatient service. Between January 2003 and December 2005, the initial diagnosis date of SA served as the index date for each patient. Patients with SA before or after the enrollment period and patients who previously experienced dementia (ICD-9-CM codes 290.0, 290.1, 290.10, 290.11, 290.12, 290.13, 290.2, 290.20, 290.21, 290.22, 290.3, 290.4, 290.40, 290.41, 290.42, 290.43, 294.1, 294.10, 294.11, 331.0, 331.1, and 331.2) were excluded. The selection criteria required that for all dementia patients, the ICD-9 code was assigned by neurologists, psychiatrists, or general practitioners, and subjects were included in our study only if they had at least two consensus ambulatory visits or one inpatient service in the follow-up periods. Finally, we excluded patients (n=3524) with a history of SA or dementia before the baseline year.

Patients were tracked for 5 years from their index dates to identify whether they developed dementia. To investigate the relationship between dementia and particular comorbidities, we also adopted several covariables, such as hypertension (ICD-9-CM 401.X-405.X), diabetes mellitus (ICD-9-CM 250.X), hyperlipidemia (ICD-9-CM 272.X), and stroke (ICD-9-CM 430.X-438.X). We included these covariables in our analytical model to examine the relationship between dementia and particular comorbidities.

Our comparison cohort was randomly sampled from the remaining patients in the LHID2005 data set. Patients were excluded if they were diagnosed with, or had a history of, any other SA or dementia. The final comparison cohort was selected from the LHID2005 data set through random selection to match a control-to-case ratio of 5 based on age, sex, and index year. All patients were followed from the index date (between January 2003 and December 2005) until they developed dementia or until the end of the 5-year follow-up period between January 2008 and December 2010.

### Level of Urbanization

We adopted the criteria published by the Taiwanese NHRI on urbanization levels in Taiwan, which include 7 strata. Level 1 represents the most urbanized communities, and Level 7 represents the least urbanized communities. All 359 regions in Taiwan were stratified into 7 levels. The standards for classification included population density (people/km^2^), the proportion of people with a college education or above, the proportion of people over 65 years of age, the proportion of agricultural workers, and the number of physicians per 100 000 people. However, because there were only small numbers of SA cases at Levels 4, 5, 6, and 7, these 4 levels were combined into a single group, hereafter referred to as Level 4. Therefore, we have 4 strata for urbanization levels.

### Statistical Analysis

We performed all data processing and statistical analyses by using the Statistical Package for Social Science (SPSS) software, Version 18 (SPSS Inc). We used Pearson x^2^ tests to compare differences in geographic location, monthly income, and urbanization level of patient residences between the study group and the comparison group. Stratified Cox proportional-hazards regression analysis (stratified according to sex, age group, and index year) was performed to examine the risk of subsequent dementia during the 5-year follow-up period for patients with and without SA. All patients were followed from the index date until they developed dementia or until the end of the 5-year follow-up period. We used hazard ratios (HRs) and 95% confidence intervals (CIs) to show the risk of dementia. We considered a 2-sided *P* value < .05 to be statistically significant.

## Results

**Figure 1:**
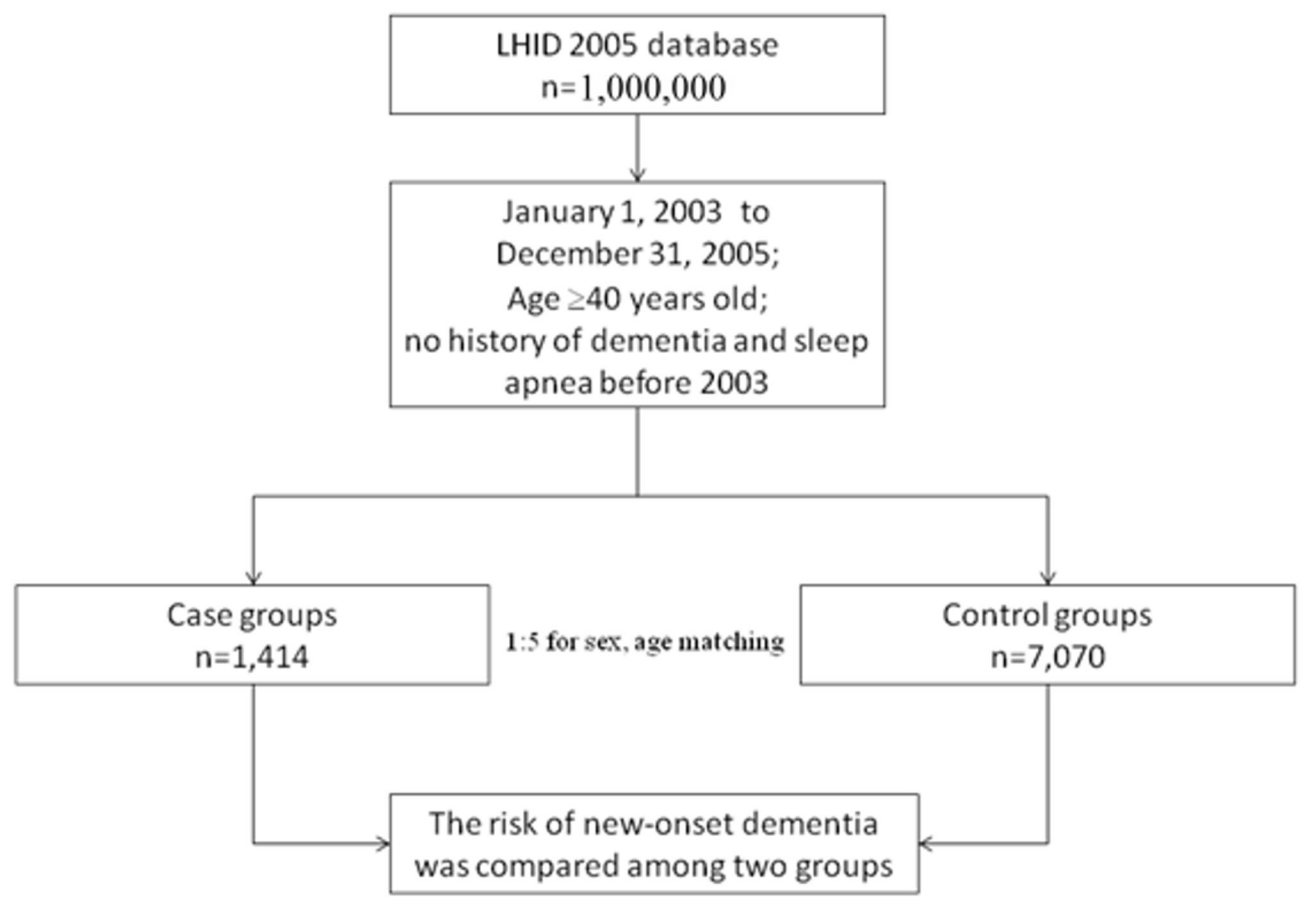
The research design followchart of present study.

**Figure 2:**
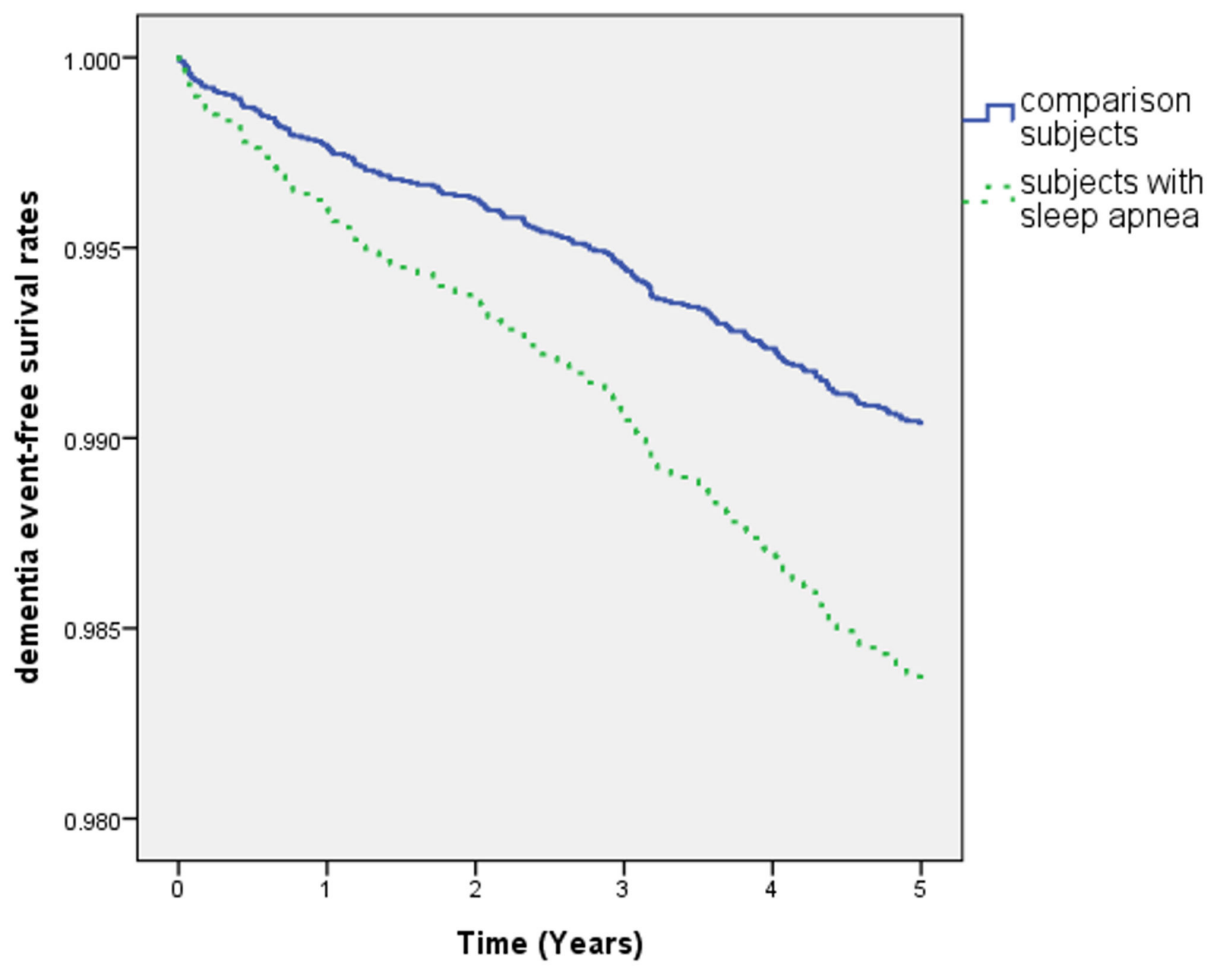
Dementia event-free risk over time among two groups.

Figure 1 shows the research design flowchart. A total of 1414 patients diagnosed with SA matched the inclusion criteria; 7070 patients were included in the comparison cohort. Table 1 shows the distributions of sociodemographic characteristics and the comorbid medical disorders for the SA and comparison groups. This table shows that SA patients suffered more hypertension (*P* < .001), hyperlipidemia (*P* < .001), diabetes (*P* < .001), stroke (*P* < .001), and had a lower monthly income (*P* < .001), than controls. More patients resided in northern Taiwan, but there was no significant difference in geographic region. During the 5-year follow-up period, 62 SA patients (4.4% of the study cohort) and 137 non-SA patients (1.9% of the comparison cohort) developed dementia. Cox regression analysis demonstrated that the crude HR of dementia was 2.32 times greater for patients with SA (95% CI =1.72–3.13) (*P* < .001) than for the comparison cohort. The HR remained significant after adjusting for potential confounders (including hypertension, hyperlipidemia, diabetes, stroke, urbanization level and monthly income variables). Table 2 shows that the adjusted HR was 1.70, 95% CI = 1.26–2.31) (*P* < .01). Cox proportional-hazards regression analysis indicated that SA patients had significantly lower 5-year dementia-free survival rates (*P* < .01), as Figure 2 shows. The number and percentage of newly developing dementia cases among both SA patients and controls stratified by age and gender are listed in Table S1. 

**Table 1:** Demographic Characteristics for the Selected Subjects, Stratified by Presence/Absence of Sleep Apnea from 2003 to 2005 (n=8484).

	Subjects with Sleep Apnea (n=1414)		Subjects without Sleep Apnea (n=7070)		P value
	n	%	N	%	
**Gender**					1
Male	839	59.3	4195	59.3	
Female	575	40.7	2875	40.7	
**Age (Years**)					1
40-49	565	40.0	2825	40.0	
50-59	437	30.9	2185	30.9	
60-69	219	15.5	1095	15.5	
Over 70	193	13.6	965	13.6	
**Follow-up, year, mean (SD)**					<0.001
	4.78	(1.11)	4.93	(0.61)	
**Urbanization level**					<0.001
1(most urbanized)	525	37.1	2417	34.2	
2	444	31.4	1980	28.0	
3	199	14.1	1070	15.1	
4(least urbanized)	246	17.4	1603	22.7	
**Monthly income**					<0.001
0	293	20.7	1377	19.5	
NT$ 1-15840	225	15.9	913	12.9	
NT$ 15841-25000	488	34.5	2861	40.5	
≧25001	408	28.9	1919	27.1	
**Geographic region**					0.06
North	702	49.6	3277	46.4	
Central	319	22.6	1755	24.8	
South	334	23.6	1677	23.7	
Eastern	59	4.2	361	5.1	
**Hypertension**					<0.001
Yes	899	63.6	3399	48.1	
No	515	36.4	3671	51.9	
**Hyperlipidemia**					<0.001
Yes	773	54.7	2644	37.4	
No	641	45.3	4426	62.6	
**Diabetes**					<0.001
Yes	507	35.9	1790	25.3	
No	907	64.1	5280	74.7	
**Stroke**					<0.001
Yes	354	25.0	1095	15.5	
No	1060	75.0	5975	84.5	

**Table 2:** Hazard Ratios of Dementia among Sleep Apnea Subjects during the 5-year Follow-up Period from the Index Ambulatory Visits or Inpatient Care from 2003 to 2005.

	Total		Patients with Sleep_apnea		Patients without Sleep_apnea	
Development of Dementia	NO.	(%)	NO.	(%)	NO.	(%)
5-year follow-up period						
Yes	199	2.3	62	4.4	137	1.9
No	8285	97.7	1352	95.6	6933	98.1
Crude HR (95% CI)				2.32 (1.72-3.13)***	1	
Adjusted HR (95% CI)				1.70 (1.26-2.31)**	1	

Total sample number =8484.

Both crude and adjusted HRs were calculated by Cox proportional hazard regressions, and stratified by age and sex.

Adjustments are made for patients’ hypertension, hyperlipidemia, diabetes, stroke, urbanization level, monthly income.

** Indicates p<0.01;*** Indicates p<0.001

We further tested whether SA is a gender-dependent, age-dependent, and time-dependent risk factor for dementia. We found that, compared with control patients, female (HR: 2.38, 95% CI =1.51–3.74; *P* < .001), but not male, SA patients were more likely to develop dementia within a 5-year follow-up period (Table S2). We divided the SA patients into 4 groups according to age ( 40-49, 50-59, 60-69, ≥ 70 ). We found that those in 50-59 group, 60-69 group, and ≥ 70 group had a greater risk of developing dementia, and the statistical significance persisted in the 50-59 group and ≥ 70 group after adjusting for potential confounders (50-59 group, adjusted HR:3.63, 95% CI =1.67–7.88; ≥ 70 group, adjusted HR:1.53, 95% CI = 1.01-2.33) (Table S3). This risk did not exist in SA patients aged 40-49 years. We then explored the age-dependent effect in the 2 genders and found males with SA aged 50-59 years had 6.08 times greater risk of developing dementia (95% CI = 1.96-18.90) (Table S4). For females with SA, the risk of dementia increased after age 50. The crude HR of dementia were all significant in the 50-59 group, 60-69 group, and ≥ 70 group, and the statistical significance persisted in ≥ 70 group after adjusting for potential confounders (adjusted HR:3.20, 95% CI =1.71–6.00) (Table S4). In separate analyses of dementia that occurred in the first and in the second 2.5 years of the follow-up, we found the increasing HRs for dementia in the first 2.5 years of follow-up in SA patients. The crude HR of dementia was 2.88, 95% CI =1.93-4.31, *P*< .0001; the adjusted HR was 2.04, 95% CI =1.35-3.07, *P*< .001 (Table S5, Figure S1). The increased risk did not exist for SA patients in the second 2.5 years of follow-up (Table S5, Figure S2). Finally, we analyzed the SA impact on the risk of developing the 2 most common forms of dementia, AD and vascular dementia, respectively. SA could increase the risk of vascular dementia only (adjust HR: 1.93, 95% CI = 1.00-3.77; *P* < .005) (Table S6).

## Discussion

This is the first longitudinal study using a large-scale nationwide database to demonstrate an increased risk of dementia for patients recently diagnosed with SA over a 5-year period. The main finding of our study is that Cox proportional hazard analysis, stratified according to patient age, gender, and year of index date, shows that the hazard ratio (HR) of dementia is 1.44 times greater for patients with SA than for the comparison group. This finding is in agreement with our hypotheses that SA patients may be at a greater risk of developing dementia. This finding is also in agreement with a recent prospective study of older women, which showed that SA patients are at a 1.85 times increased risk of minimal cognitive impairment or dementia 5 years later [8]. Separate analyses showed SA is an age-dependent and time-dependent risk factor for developing dementia. Furthermore, our study confirmed, in SA subjects, the prevalence of hypertension, hyperlipidemia, diabetes, stroke and lower income exceeds that seen in the control subjects, which was consistent with the results of previous studies [9]. All these strengthen the reliability of our findings.

Although these findings support our hypothesis that SA is associated with increased risk of dementia, they also suggest the existence of an association that may not be causal in nature. Several possible explanations for these results data should be considered. First, a study by Yaffe et al [8] shows that hypoxia, but not sleep fragmentation or sleep duration, was associated with mild cognitive impairment or dementia in SA patients. This finding is in agreement with animal studies showing that, in rodents, exposure to intermittent hypoxia, a hallmark of SA, is associated with neurobehavioral impairment and increased apoptosis in the hippocampus [10]. Furthermore, voxel-based morphometry has shown a gray matter reduction in brain regions that regulate memory and executive functions (ie, frontal, parietal and temporal areas, and hippocampus) [11,12]. These findings suggest that intermittent hypoxia in SA may play a major role in cognitive dysfunction and gray matter reduction, which in turn may contribute to the development of dementia. Second, mice subjected to hypoxic conditions unambiguously demonstrated upregulation in cerebral amyloid plaque formation and tau phosphorylation, as well as memory deficit [13]. Beta-amyloid deposition and tau phosphorylation in the brain are common features of Alzheimer’s disease (AD), which may contribute to the link between SA and dementia. Third, both SA and dementia are complex diseases that involve complex genetic and environmental interactions. Recent studies have shown that the apolipoprotein epsilon 4 (APOE4) allele increases the risk of SA, particularly that which is obstructive in mechanism [14]. The APOE4 allele is associated with cognitive decline and the development of dementia in the general population. These findings suggest a shared genetic background between SA and dementia. In addition, O’Hara et al [15] showed that SA interacts with the APOE4 allele to affect memory negatively, suggesting that APOE4 carriers with SA may be at a higher risk of developing dementia. Fourth, it is conceivable that vascular risk factors are more prevalent in SA patients than in the general population [16,17], and that the incidence of dementia increases in SA patients.

SA has a male predominance and, compared with their male counterparts, female patients experience SA at an older age and with higher body-mass-index [18]. Among SA population, women has been found to have more somatic symptoms than men, such as fatigue, morning headaches, insomnia, depression and use of sedatives [19]. The second main finding of this study is that, female, but not male, SA patients were more likely than control patients to develop dementia within a 5-year follow-up period (Table S2). This finding suggests that SA is a gender-dependent risk factor for dementia. To our knowledge, there is no clinical research showing a gender effect of SA on cognitive dysfunction. Study by Macey et al [20] had showed that gender differences in white matter structural integrity appeared in SA patients, with females more affected than males. They suggested that these female-specific structural changes may contribute to or derive from neuropsychological and physiological symptom differences between sexes. However, animal studies had shown that male, but not female, rats exposed to intermittent hypoxia exhibited working memory deficits [21]. Additionally, Sholl analysis of Golgi-stained neurons revealed decreased dendritic branching in the frontal cortex, but not the hippocampus, of male but not female rats exposed to intermittent hypoxia [21]. These animal studies are in contrast with our clinical findings that female SA patients are more likely to develop dementia, the findings of both studies suggest gender differences in SA-associated cognitive deficits.

The findings from our national population-based cohort study provide support for the link between SA and dementia. Although few treatments are available for dementia, highly effective treatments are available for SA. Studies on SA treatment have indicated that continuous positive airway pressure (CPAP) therapy can be effective in reducing SA events and their associated cognitive and affective sequelae [15,22]. 

Our findings show that females with SA have a higher risk of dementia from the age of 50, and those aged 70 years or above have the greatest risk. Our findings suggest that the detrimental effect of SA may be present till the advanced post-menopause age in the female population. The age range for menopause is generally from 40–60 years, with a median of 49 to 52 years, depending on the population [23]. The intensive sex hormonal decreases that accompany menopause have a profound and permanent physiologic effect. Female hormones have been reported to increase the activity of dilator upper-airway muscles [24], and the ventilatory response to hypercapnia and hypoxia [25]. Pickett et al [26] reported that combined estrogen and progesterone treatment reduced the number of apnea events per night from 15 to 3 in healthy postmenopausal women. Saaresranta et al. [27] also found that estrogen therapy predicted the better nocturnal oxyhemoglobin saturation in healthy postmenopausal women. Taken together, female hormones may decrease the frequency and severity of SA, protect against SA-induced hypoxia, and have favorable respiratory effects. Since female hormone may decrease with age in women [28], its beneficial effects may completely disappear at an advanced age, and the detrimental impact of SA on cognition may then be present in female aged above 70 years. Future studies need to clarify the underlying mechanisms of increased risk of dementia in aged women with SA.

Yaffe et al. [8] studied nearly 300 older women from a large community-dwelling cohort and found that SA was a risk factor for the cognitive impairment at approximately 5-year of follow-up. Based on our study results, the follow-up period could be shortened to 2.5 years for evaluating the SA impact on the dementia susceptibility (Table S5, Figure S1). Moreover, in this study, we enrolled patients only with newly diagnosed SA, and the follow-up period may represent the duration of SA. This in turn expanded our ability to assess the prospective relationship and the exact duration between the initial onset of SA and the development of dementia. The first few years of SA may be a critical period for developing cognitive deficit and for intervention. However, the fact that an association between SA and dementia exists only in the first 2.5 years but not in the second 2.5 years strengthens the conjecture that this could be a case of reverse causality. Future longitudinal cohort studies are needed to clarify such reversed or reciprocal causality of SA and dementia.

We finally analyzed the SA impact on the risk of the 2 most common forms of dementia, AD and vascular dementia, respectively. The adjusted HR for AD is even higher than for vascular dementia, but the association does not reach significance for AD due to the small number of cases (Table S6). The findings suggest that the association with SA is of similar strength for both illnesses. 

The strength of this study is that it is a matched control cohort study design, which considers many variables to minimize potential confounding factors. Because the National Health Insurance in Taiwan is a single-payer, mandatory health insurance program with affordable payments, the majority of events could be traced and referral bias minimized. Nevertheless, there are still some limitations to this study. First, patient enrollment based on administrative claims data may be relatively inaccurate, which is a weak point inherent in all database research. Thus, the accuracy of the dementia diagnoses in this type of research might be questionable. A study by Taylor et al [29] using 5 years of claims data demonstrated that approximately 75% of AD or AD-related disorders were identified. The sensitivity of claims data in detecting dementia increases with the severity of cognitive impairment, suggesting that studies involving claims data might have missed a higher proportion of those with milder forms of dementia. Regarding the diagnosis of SA, most of the sleep centers in Taiwan follow the guidelines of the AASM (American Academy of Sleep Medicine) for the diagnosis of SA. These are based on both clinical symptoms and a standard overnight polysomnography study.

Second, the NHI database includes only patients who sought treatment for SA and dementia, but does not include important parameters such as education, family history, tobacco smoking status, body mass index, and clinical severity. Thus the danger of confounding and differential misclassification is particularly great. Further research is necessary to clarify the effects of these factors. Finally, the severity of SA cannot be determined based on our database. In addition, the treatment status and compliance for CPAP, a major treatment for SA patients, cannot be determined based on the registry.

In conclusion, our study supports the hypothesis that patients with SA are at a higher longitudinal risk of developing dementia. SA may be a gender-dependent, age-dependent, and time-dependent risk factor for dementia. However, the specific mechanisms that underlie this association remain unknown, and further study is necessary to confirm our findings and explore underlying pathomechanisms.

## Supporting Information

Table S1 **The Number and Percentage of Newly Developing Dementia in both SA Patients and Controls Stratified by Age and Gender.**
(DOCX)

Table S2 **Hazard Ratios for Dementia among Subjects with Sleep Apnea (Case) and the Comparison Cohort (Control) by Gender Group.**
(DOCX)

Table S3 **Hazard Ratios for Dementia among Subjects with Sleep Apnea (Case) and the Comparison Cohort (Control) by Age Group.**
(DOCX)

Table S4 **Hazard Ratios for Dementia among Subjects with Sleep Apnea (Case) and the Comparison Cohort (Control) by Age Group in Different Gender.**
(DOCX)

Table S5 **Hazard Ratios for Dementia among Subjects with Sleep Apnea (Case) and the Comparison Cohort (Control) by Different Follow-up Period.**
(DOCX)

Table S6 **Hazard Ratios for Dementia Subtype among Subjects with Sleep Apnea (Case) and the Comparison Cohort (Control).**
(DOCX)

Figure S1 **Dementia event-free risk over first 2.5 years of follow-up.**
(TIF)

Figure S2 **Dementia event-free risk over second 2.5 years of follow-up.**
(TIF)
