# Supplementary material for: Sleep Apnea and the Risk of Dementia: A Population-Based 5-Year Follow-Up Study in Taiwan
Source: PLoS One. 2013 Oct 24;8(10):e78655. doi: 10.1371/journal.pone.0078655 (PMC3813483; doi:10.1371/journal.pone.0078655)
Supplement: Table S1 — The Number and Percentage of Newly Developing Dementia in both SA Patients and Controls Stratified by Age and Gender. (DOCX) [file pone.0078655.s001.docx]

| **Table S1** The Number and Percentage of Newly Developing Dementia in both SA Patients and Controls Stratified by Age and Gender | | | | | | | |
| --- | --- | --- | --- | --- | --- | --- | --- |
|  | Sleep apnea subjects | | |  | Non-sleep apnea subjects | | |
|  | Dementia |  | Non-dementia |  | Dementia |  | Non-dementia |
|  | n (%) |  | n (%) |  | n (%) |  | n (%) |
| Age |  |  |  |  |  |  |  |
| 40-49 | 5 (8.1) |  | 560 (41.4) |  | 9 (6.6) |  | 2816 (40.6) |
| 50-59 | 13 (21.0) |  | 424 (31.4) |  | 14 (10.2) |  | 2171 (31.3) |
| 60-69 | 12 (19.4) |  | 207 (15.3) |  | 29 (21.2) |  | 1066 (15.4) |
| 70- | 32 (51.6) |  | 161 (11.9) |  | 85 (62.0) |  | 880 (12.7) |
| Gender |  |  |  |  |  |  |  |
| Male | 31 (50.0) |  | 808 (59.8) |  | 84 (61.3) |  | 4111 (59.3) |
| Female | 31 (50.0) |  | 544 (40.2) |  | 53 (38.7) |  | 2822 (40.7) |
