# Supplementary material for: Sleep Apnea and the Risk of Dementia: A Population-Based 5-Year Follow-Up Study in Taiwan
Source: PLoS One. 2013 Oct 24;8(10):e78655. doi: 10.1371/journal.pone.0078655 (PMC3813483; doi:10.1371/journal.pone.0078655)
Supplement: Table S2 — Hazard Ratios for Dementia among Subjects with Sleep Apnea (Case) and the Comparison Cohort (Control) by Gender Group. (DOCX) [file pone.0078655.s002.docx]

| **Table S2** Hazard Ratios for Dementia among Subjects with Sleep Apnea (Case) and the Comparison Cohort (Control) by Gender Group | | | | | | | |
| --- | --- | --- | --- | --- | --- | --- | --- |
|  | Gender Group | | | | | | |
| Presence of Dementia | Female | | |  | Male | | |
|  | Case |  | Control |  | Case |  | Control |
|  | n (%) |  | n (%) |  | n (%) |  | n (%) |
| Yes | 31 (5.4) |  | 53 (1.8) |  | 31 (3.7) |  | 84 (2.0) |
| Crude HR (95% CI) | 3.00 (1.93-4.68)*** |  | 1 |  | 1.89 (1.25-2.85)** |  | 1 |
| Adjusted HR (95%CI) | 2.38 (1.51-3.74)*** |  | 1 |  | 1.26 (0.83-1.92) |  | 1 |

Adjustments are made for hypertension, hyperlipidemia, diabetes, stroke, urbanization level, monthly income.

** Indicates p<0.01; *** Indicates p<0.001
