# Supplementary material for: Sleep Apnea and the Risk of Dementia: A Population-Based 5-Year Follow-Up Study in Taiwan
Source: PLoS One. 2013 Oct 24;8(10):e78655. doi: 10.1371/journal.pone.0078655 (PMC3813483; doi:10.1371/journal.pone.0078655)
Supplement: Table S3 — Hazard Ratios for Dementia among Subjects with Sleep Apnea (Case) and the Comparison Cohort (Control) by Age Group. (DOCX) [file pone.0078655.s003.docx]

**Table S3** Hazard Ratios for Dementia among Subjects with Sleep Apnea (Case) and the Comparison Cohort (Control) by Age Group

| Development of Dementia | Age Group | | | | | | | | | | | | | | |  |  |
| --- | --- | --- | --- | --- | --- | --- | --- | --- | --- | --- | --- | --- | --- | --- | --- | --- | --- |
|  | 40-49 | | |  | 50-59 | | |  | 60-69 | | |  | ≥70 or older | | | |  |
|  | Case |  | Control |  | Case |  | Control |  | Case |  | Control |  | Case |  | Control | | |
|  | n (%) |  | n (%) |  | n (%) |  | n (%) |  | n (%) |  | n (%) |  | n (%) |  | n (%) | | |
| Yes | 5 (0.9) |  | 9 (0.3) |  | 13 (3.0) |  | 14 (0.6) |  | 12 (5.5) |  | 29 (2.6) |  | 32 (16.6) |  | 85 (8.8) | | |
| Crude HR (95% CI) | 2.80 (0.94-8.35) |  | 1 |  | 4.76 (2.24-10.12)*** |  | 1 |  | 2.12 (1.08-4.15)* |  | 1 |  | 2.04 (1.34-3.06)** |  | 1 | | |
| Adjusted HR (95%CI) | 1.77 (0.56-5.56) |  | 1 |  | 3.63 (1.67-7.88)** |  | 1 |  | 1.73 (0.88-3.43) |  | 1 |  | 1.53 (1.01-2.33)* |  | 1 | | |

Adjustments are made for patients’ hypertension, hyperlipidemia, diabetes, stroke, urbanization level, monthly income.

* Indicates p<0.05;** Indicates p<0.01; *** Indicates p<0.001
