# Supplementary material for: Sleep Apnea and the Risk of Dementia: A Population-Based 5-Year Follow-Up Study in Taiwan
Source: PLoS One. 2013 Oct 24;8(10):e78655. doi: 10.1371/journal.pone.0078655 (PMC3813483; doi:10.1371/journal.pone.0078655)
Supplement: Table S4 — Hazard Ratios for Dementia among Subjects with Sleep Apnea (Case) and the Comparison Cohort (Control) by Age Group in Different Gender. (DOCX) [file pone.0078655.s004.docx]

| **Table S4** Hazard Ratios for Dementia among Subjects with Sleep Apnea (Case) and the Comparison Cohort (Control) by Age Group in Different Gender | | | | | | | | | | | | | | | | |  |
| --- | --- | --- | --- | --- | --- | --- | --- | --- | --- | --- | --- | --- | --- | --- | --- | --- | --- |
| Development of Dementia |  | Age Group | | | | | | | | | | | | | | | |
|  |  | 40-49 | | |  | 50-59 | | |  | 60-69 | | |  | ≥70 | | | |
|  |  | Case |  | Control |  | Case |  | Control |  | Case |  | Control |  | Case |  | Control | |
| Male |  | n (%) |  | n (%) |  | n (%) |  | n (%) |  | n (%) |  | n (%) |  | n (%) |  | n (%) | |
|  | Yes | 3 (0.8) |  | 6 (0.3) |  | 8 (3.3) |  | 6 (0.5) |  | 5 (4.5) |  | 16 (2.9) |  | 15 (13.3) |  | 56 (9.9) | |
|  | Crude HR (95% CI) | 2.53 (0.63-10.10) |  | 1 |  | 6.88 (2.39-19.82)*** |  | 1 |  | 1.58 (0.58-4.31) |  | 1 |  | 1.41 (0.80-2.49) |  | 1 | |
|  | Adjusted HR (95%CI) | 1.90 (0.45-8.11) |  | 1 |  | 6.08 (1.96-18.90)** |  | 1 |  | 0.96 (0.34-2.70) |  | 1 |  | 0.91 (0.50-1.63) |  | 1 | |
|  |  |  |  |  |  |  |  |  |  |  |  |  |  |  |  |  | |
| Female | Yes | 2 (1.0) |  | 3 (0.3) |  | 5 (2.6) |  | 8 (0.8) |  | 7 (6.5) |  | 13 (2.4) |  | 17 (21.3) |  | 29 (7.3) | |
|  | Crude HR (95% CI) | 3.34 (0.56-19.98) |  | 1 |  | 3.17 (1.04-9.69)* |  | 1 |  | 2.78 (1.11-6.98)* |  | 1 |  | 3.28 (1.80-5.97)*** |  | 1 | |
|  | Adjusted HR (95%CI) | 2.44 (0.32-18.88) |  | 1 |  | 1.92 (0.58-6.28) |  | 1 |  | 2.48 (0.97-6.34) |  | 1 |  | 3.20 (1.71-6.00)*** |  | 1 | |

*Total sample number =8484.

Both crude and adjusted HRs were calculated by stratified Cox proportional

Adjustments are made for patients’ Hypertension, Hyperlipidemia, Diabetes, Stroke, Urbanization level, Monthly income.

* Indicates p<0.05, ** Indicates p<0.01, *** Indicates p<0.001
