# Supplementary material for: Sleep Apnea and the Risk of Dementia: A Population-Based 5-Year Follow-Up Study in Taiwan
Source: PLoS One. 2013 Oct 24;8(10):e78655. doi: 10.1371/journal.pone.0078655 (PMC3813483; doi:10.1371/journal.pone.0078655)
Supplement: Table S5 — Hazard Ratios for Dementia among Subjects with Sleep Apnea (Case) and the Comparison Cohort (Control) by Different Follow-up Period. (DOCX) [file pone.0078655.s005.docx]

| **Table S5** Hazard Ratios for Dementia among Subjects with Sleep Apnea (Case) and the Comparison Cohort (Control) by Different Follow-up Period | | | | | | | |
| --- | --- | --- | --- | --- | --- | --- | --- |
|  | For 5 years of the follow-up | | | | | | |
| Presence of dementia | First 2.5 years of the follow-up | | |  | Second 2.5 years of the follow-up | | |
|  | Case |  | Control |  | Case |  | Control |
|  |  |  |  |  |  |  |  |
| Crude HR (95% CI) | 2.88 (1.93-4.31)*** |  | 1 |  | 1.35 (0.85-2.14) |  | 1 |
| Adjusted HR (95%CI) | 2.04 (1.35-3.07)** |  | 1 |  | 0.99 (0.62-1.57) |  | 1 |

Adjustments are made for patients’ monthly income, urbanization level, hypertension, hyperlipidemia, diabetes, stroke

** Indicates p<0.01; *** Indicates p<0.001
