# Supplementary material for: Sleep Apnea and the Risk of Dementia: A Population-Based 5-Year Follow-Up Study in Taiwan
Source: PLoS One. 2013 Oct 24;8(10):e78655. doi: 10.1371/journal.pone.0078655 (PMC3813483; doi:10.1371/journal.pone.0078655)
Supplement: Table S6 — Hazard Ratios for Dementia Subtype among Subjects with Sleep Apnea (Case) and the Comparison Cohort (Control). (DOCX) [file pone.0078655.s006.docx]

| **Table S6** Hazard Ratios for Dementia Subtype among Subjects with Sleep Apnea (Case) and the Comparison Cohort (Control). | | | | | | | |  |
| --- | --- | --- | --- | --- | --- | --- | --- | --- |
|  | | | | | | | | |
| Development of Dementia | | Dementia categories | | | | | | |
|  |  | Alzheimer's disease | | |  | Vascular dementia | | |
|  |  | Case |  | Control |  | Case |  | Control |
|  |  | n (%) |  | n (%) |  | n (%) |  | n (%) |
| Yes | | 2 (0.14) |  | 4 (0.05) |  | 14 (0.99) |  | 23 (0.32) |
| Crude HR (95% CI) | | 2.50 (0.46-13.65) |  | 1 |  | 3.06 (1.57-5.95) ** |  | 1 |
| Adjusted HR (95%CI) | | 2.40 (0.43-13.42) |  | 1 |  | 1.93 (1.00-3.77) * |  | 1 |

Adjustments are made for patients’ monthly income, urbanization level, hypertension, hyperlipidemia, diabetes, stroke

* Indicates p<0.05; ** Indicates p<0.01
