# Supplementary figures and images for: Sleep Apnea and the Risk of Dementia: A Population-Based 5-Year Follow-Up Study in Taiwan
Source: PLoS One. 2013 Oct 24;8(10):e78655. doi: 10.1371/journal.pone.0078655 (PMC3813483; doi:10.1371/journal.pone.0078655)

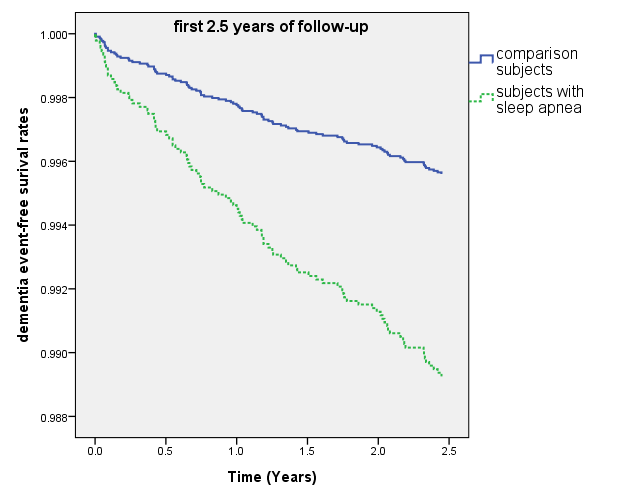

Supplement: Figure S1 — Dementia event-free risk over first 2.5 years of follow-up. (TIF) [file pone.0078655.s007.tif]

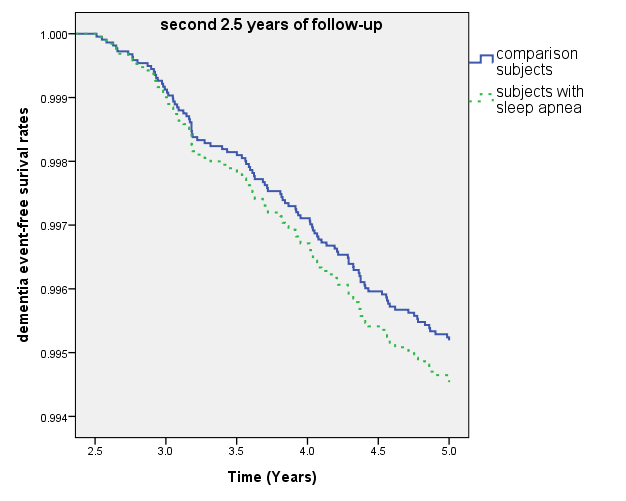

Supplement: Figure S2 — Dementia event-free risk over second 2.5 years of follow-up. (TIF) [file pone.0078655.s008.tif]
